# Yeast Biodiversity of Karst Waters: Interest of Four Culture Media and an Improved MALDI-TOF MS Database

**DOI:** 10.1007/s00248-023-02336-1

**Published:** 2024-01-04

**Authors:** Clément Grandhay, Emma Prétot, Victor Klaba, Hélène Celle, Anne-Cécile Normand, Xavier Bertrand, Frédéric Grenouillet

**Affiliations:** 1 Université de Franche-Comté, CHU Besançon, CNRS, Chrono-environnement, F-25000 Besançon, France; 2 Université de Franche-Comté, CNRS, Chrono-environnement, F-25000 Besançon, France; 3 AP-HP, Groupe Hospitalier La Pitié-Salpêtrière, Service de Parasitologie Mycologie, 75013 Paris, France

**Keywords:** Yeast, Karst, Water, Culture media, MALDI-TOF mass spectrometry

## Abstract

Karst aquifers are a significant source of drinking water and highly vulnerable to pollution and microbial contamination. Microbiological regulations for the quality of drinking water mostly focus on bacterial levels and lack guidance concerning fungal contamination. Moreover, there is no standardised microbial analysis methodology for identifying fungi in water. Our main objective was to establish the most effective culture and identification methodology to examine yeast diversity in karst waters. We assessed the comparative efficacy of four culture media (CHROMagar Candida, dichloran glycerol 18% [DG18], dichloran rose Bengal chloramphenicol [DRBC], and SYMPHONY agar) for yeast isolation from karst water samples. Furthermore, we investigated the comprehensiveness of databases used in MALDI-TOF mass spectrometry (MALDI-TOF MS) for identifying environmental yeast species. In total, we analysed 162 water samples, allowing the identification of 2479 yeast isolates. We demonstrate that a combination of four culture media, each with distinct specifications, more efficiently covers a wide range of yeast species in karst water than a combination of only two or three. Supplementation of a MALDI-TOF MS database is also critical for analysing environmental microbial samples and improved the identification of yeast biodiversity. This study is an initial step towards standardising the analysis of fungal biodiversity in karst waters, enabling a better understanding of the significance of this environmental reservoir in relation to public health.

**Supplementary Information:**

The online version contains supplementary material available at 10.1007/s00248-023-02336-1.

## Introduction

Karst groundwater is a significant source of drinking water worldwide and is believed to serve approximately 25% of the world’s population [1]. Water of karst systems is particularly vulnerable to a wide range of chemical and biological pollutants, which have a direct impact on the quality of drinking water [2]. As a result, water from karst aquifers represents a potential risk of exposure to contaminants for the human population, and, therefore, drinking water regulations are essential. Currently, the microbiological analysis of drinking water focuses primarily on bacterial parameters that indicate faecal contamination. Drinking water safety and quality regulations are heavily based on the presence of *Escherichia coli*, enterococci, coliforms, and clostridia [3, 4]. The inclusion of fungi in current national or international drinking water regulations is practically non-existent, although their presence in water has been well established over the last 30 years [5, 6]. The Swedish National Food Administration is the only legislation that includes a parametric value for fungi in drinking water [7].

The literature on the biodiversity of fungi in karst waters is scarce compared to that on bacteria. Several recent studies have characterised the fungi found in drinking water, but most focused mainly on filamentous fungi, and little attention has been accorded to yeast in water [8–10]. These studies have used culture methods to fungi in water, using different isolation media with various incubation times and temperatures. The heterogeneity of isolation procedures has been highlighted previously [5, 10]. High nutrient media are often used, such as Sabouraud dextrose agar and malt extract agar, but are quickly covered by fast-growing moulds [8–10]. Alternatively, more selective media containing fungicides that inhibit overgrowth of these fast-growing moulds, such as dichloran-18% glycerol agar (DG18) or dichloran-Rose Bengal agar (DRBC) [6, 11], were used. The use of different media may result in selectivity for some fungal species and loss of others. Most of the studies used a single medium, sometimes two, and have mainly reported the presence of moulds in the water and, sporadically, of yeasts. [6, 11]. Thus, many aspects concerning the biodiversity of yeast in drinking water are still poorly understood. Currently, there are no international guidelines for the analysis of fungi in drinking water. The lack of a standardised protocol makes it difficult to compare studies and ensure reliable results due to significant differences in methodology [11]. To date, there is a lack of studies evaluating the ability of different culture media to characterise fungal biodiversity in water as effectively as possible, especially for yeasts.

There is an urgent need to define a standard protocol for the analysis of fungal biodiversity in water. The current study is part of the TRANSKARST project, a transdisciplinary study of karst waters in France that was launched in 2019. The primary endpoint of this study is to identify the best culture strategy for yeast in karst waters using a combination of four culture media. Second, we assessed the importance of complementing and combining existing MALDI-TOF MS databases to improve the identification of environmental yeasts. This has enabled us to identify a very high level of fungal biodiversity in these waters, which has not been previously reported.

## Materials and Methods

### Study Sites and Sample Collection

The study area is the recharge area of the Arcier spring (102 km^2^) located in the Jura Mountains (Eastern France). This spring (ARC; 47.2673538, 6.1209294) is the outlet of a complex karst system, extending mainly on the Saône plateau. Two main swallow holes allow surface water to enter the underground and contribute to groundwater circulation: the Creux-sous-Roche (CRE) and Nancray swallow holes (PER). Near the town of Saône (47.233333, 6.116667), the CRE drains the western part of the plateau, collecting water from (1) the Saône swamp (800 ha) and (2) the FSA stream via the PSC spring. The PER is located near the town of Nancray (47.2448, 6.1806) and drains the waters of the RUI spring, as well as the eastern part of the plateau. Mapping and schematic representation of the Arcier karst watershed are available elsewhere [12]. The Arcier spring is crucial in terms of water quality and quantity at the local scale because it is the main drinking water supply for the city of Besançon (118,000 inhabitants).

Water samples were collected in sterile Falcon flasks once or twice a month from June 2020 to November 2021 at each of the six selected sampling sites (ARC, CRE, FSA, PER, PSC, and RUI). Samples were transported at +4 °C and filtered on the day of collection. Due to logistical constraints, some sampling sessions could not be carried out. In seven sampling sessions, PSC was not sampled because of flooding.

### Culture Media

Four different culture media were used during the study. Their choice was based on their capacity of isolation of yeast and/or limitation of filamentous overgrowth. DG18 and DRBC media (BIOKAR, Allonne, France) inhibit filamentous growth due to the presence of dichloran, as previously described [12]. CHROMagar Candida medium (Graso, Starogard Gdanski, Poland) facilitates the macromorphological differentiation of yeast colonies belonging to different species [13]. SYMPHONY medium (BIOKAR, Allonne, France) has been recently developed for the analysis of environmental yeasts and moulds and the analysis of membrane-filtered water. We evaluated the performance of each media alone or in combination of two or three, compared with the combination of four media. We arbitrarily considered that the combination of the four media allowed the development of all the yeasts.

### Sample Filtration and Yeast Isolation

Yeast isolation procedure was based on the Swedish standard method for microfungal analysis in water [14]. Volumes of 100 mL of the water samples were filtered through 0.45 μm cellulose membranes (EZ-Fit™ filtration units, Millipore, Molsheim, France) by vacuum filtration. The membranes were then placed on 90 mm petri dishes with DG18, CHROMagar, DRBC, or SYMPHONY agar medium and incubated at 30 °C for 7 days. One sterile filtration unit was used per sampling location, and a different membrane was deposited on each agar plate. Yeast colonies were counted after 20 to 24 h (day 1) and 40 to 44 h (day 2) of incubation for the CHROMagar, DRBC, and SYMPHONY media and the plates then checked daily for new isolates until day 7 or complete overgrowth by moulds. For the DG18 media, the colony count was performed after incubation of 40 to 44 h (day 2) and 60 to 66 h (day 3), and the plates then checked for new isolates until day 7 or complete overgrowth by moulds. Macromorphological criteria allowed the differentiation of yeast colonies belonging to different species. Colony forming units (CFUs) were quantified and individual strains isolated and subcultured on fresh petri dishes with Sabouraud dextrose agar. These were incubated at 30 °C for 2 to 3 days, until identification. All yeast strains were stored in cryogenic vials at −80 °C.

### MALDI-TOF Mass Spectrometry

Yeast identification was performed primarily by matrix-assisted laser desorption ionisation coupled to time-of-flight mass spectrometry (MALDI-TOF MS) using a Bruker microflex® LRF instrument (Bruker Daltonics, Bremen, Germany). Initially, each yeast colony was analysed using a rapid MALDI-TOF MS protocol with a rapid formic acid extraction performed on subcultures after 48 h of incubation. According to the protocol of the manufacturer, a thin layer of the colony was applied to a spot on the MALDI steel plate, followed by the addition of 1 μL formic acid (70%) and then 1 μL HCCA matrix, before starting the MALDI run. This short protocol was repeated if the previous one was unsuccessful. Subsequently, a long MALDI-TOF MS protocol with preliminary formic acid/acetonitrile protein extraction [15] was performed to analyse the samples that remained unidentified after the two short protocols, and repeated one time if necessary. Thus, two rapid protocols, followed by two long protocols, were performed before yeast isolates were considered as unidentified. Final spectral data were initially compared to the MSI-2 database (https://msi.happy-dev.fr/) and then to the Bruker Biotyper Compass reference library. According to the manufacturer’s instructions, species-level identification was considered if the Bruker library LogScore was ≥1,7 and/or if the MSI-2 database score was between 20 and 100. The strains were identified using rRNA single gene barcoding marker if there was a mismatch between the MSI-2 database and the Bruker library or if they failed to be identified by either MS database.

### DNA Extraction

DNA was extracted from the yeast that remained unidentified or misidentified after the MALDI-TOF MS assays using the Chelex DNA extraction method [16]. A loopful of a yeast colony was suspended in 600 μL sterile water and 200 μL Chelex resin and incubated at 100 °C for 30 min. The yeast suspensions were then vortexed briefly and centrifuged at 14,000 rpm for 10 min. The supernatants were collected and diluted 1:10 in sterile water. A volume of 2 μL of DNA extract was used immediately for PCR. The remainder of the DNA extract was stored at −20 °C.

### Amplification and Sequencing of rRNA Genes

DNA extracts were amplified by PCR in a 50-μL volume containing 2 μL 1:10 DNA extract, 5 μL each of 3 µmol/L primers, and 25 μL FastStart PCR Master Mix (Roche, Basel, Switzerland). The internal transcribed spacer (ITS) region of the ribosomal RNA (rRNA) gene was amplified using forward primer ITS5 (5′-GGAAGTAAAAGTCGTAACAAGG-3′) and reverse primer ITS4 (5′-GCATATCAATAAGCGGAGGA-3′). In the event of unsuccessful ITS amplification, the D1/D2 region of the 26S rRNA gene was amplified using universal primers, such as the NL1 forward primer (5′-GCATATCAATAAGCGGAGGAAAAG-3′) and NL4 reverse primer (5′-GGTCCGTGTTTCAAGACGG-3′). PCR amplicons were separated by gel electrophoresis on 2% agarose gels in 1X Tris-acetate-EDTA (TAE), visualised using ethidium bromide under UV transillumination. Then, these were purified and diluted to obtain a DNA concentration between 20 and 40 μmol/L. DNA concentration was quantified on a NanoDrop instrument (Thermo Fisher Scientific, USA). PCR amplicons were sequenced using the reverse primer amongst ITS4 and NL4 at 10 µmol/L. Yeast identifications were obtained by first comparing the sequences using the Westerdijk Fungal Biodiversity Institute pairwise alignment (https://wi.knaw.nl/Pairwise_alignment) and then the GenBank Nucleotide BLAST database (https://blast.ncbi.nlm.nih.gov/Blast). According to the literature, the taxonomic threshold used for species level identification is 98.41% for the ITS and 99.51% for NL [17]. In the event that identification at the species level was unsuccessful, identification at the genus level was retained using a taxonomic threshold of 96.31% for the ITS and 97.11% for NL [17].

### Unidentified Strains

“*Lost strains*” were strains that died before identification. “*Unknown strains*” were those that remained unidentified after both MALDI-TOF MS and PCR assays.

### Supplementation of the MSI Database

The strains that were not identified by MALDI-TOF MS but successfully identified by PCR were thawed at room temperature and subcultured on both Sabouraud dextrose agar and CHROMagar. A long MALDI-TOF MS protocol, as previously described, was run on each strain from both CHROMagar and Sabouraud agar in quadruplicate. The spectral data obtained were assigned to the appropriate species and then submitted to the MSI-2 database curator for inclusion.

### Filamentous Growth Quantification

Filamentous colonies were quantified twice daily (respectively after 24, 42, 48, 66, 72, 90, 96, 120, 144 et 168 hours of incubation) during the seven-day incubation period. Each primary agar plate was assigned a percentage cellulose membrane invasion value relative to a clean membrane surface.

### Statistics

Ordinary one-way analysis of variance (ANOVA) tests followed by Tukey’s multiple comparison test were used to compare the performance of the media combinations. The *⍺* value was set to 0.05. Statistical analysis was performed, and the figure generated using GraphPad Prism version 9.5.0 (GraphPad Software, Boston, USA).

## Results

In total, 162 water samples were collected during the study, representing 29 sampling sessions. All the seasons were covered to obtain the best possible representation of biodiversity, which is potentially influenced by physical and chemical factors, depending on the climatic condition. A total of 2479 yeast isolates were analysed during the study, corresponding to 1485 identified strains, with an average of 9.2 strains identified per sample.

### Biodiversity

From a pool of 2479 yeast isolates analysed during the study, 2389 isolates (96.4%), representing 1485 strains, were identified. Among them, 2339 isolates, representing 1449 strains, were identified at the species level, and belonged to 142 different species. The remaining 50 isolates, representing 36 strains, were identified at the genus level and belonged to 10 different genera. There was a large disparity in the number of strains of each species. Indeed, three species (*Pichia fermentans*, *Metschnikowia pulcherrima*, *Hanseniaspora uvarum*) accounted for 25% of all strains identified and 30 for almost 75% of all isolated strains (Table 1). A list with all the species identified during the study is given in supplementary material (Table S1).

**Table 1:** Most common species identified in water samples compared to their frequency of isolation on each medium

Rank	Species	n^(*)^	CH^(1)^	DG^(2)^	DR^(3)^	SY^(4)^	n_i_^(**)^	MSI-2_(i)_	MSI-2_(s)_	BRU
1	*Pichia fermentans*	103	62	30	57	48	246	**•**	**•**	**•**
2	*Metschnikowia pulcherrima*	90	42	64	36	43	243	**•**	**•**	**•**
3	*Hanseniaspora uvarum*	84	40	51	61	47	226	**•**	**•**	**•**
4	*Rhodotorula mucilaginosa*	72	28	28	26	28	119	**•**	**•**	**•**
5	*Candida pseudolambica*^(***)^	57	29	2	27	25	87		**•**	
6	*Saccharomyces cerevisiae*	52	23	17	25	21	93	**•**	**•**	**•**
7	*Pichia kudriavzevii*	51	23	15	16	13	77	**•**	**•**	**•**
8	*Nakazawaea holstii*	47	19	9	23	19	80		**•**	**•**
9	*Wickerhamomyces anomalus*	42	11	18	18	12	66	**•**	**•**	**•**
10	*Nakaseomyces glabratus*	42	13	12	17	17	65	**•**	**•**	**•**
11	*Debaryomyces hansenii*	41	4	32	3	8	52	**•**	**•**	**•**
12	*Nakazawaea wyomingensis*	36	7	10	16	10	52		**•**	
13	*Clavispora lusitaniae*	35	9	10	15	11	48	**•**	**•**	**•**
14	*Pichia kluyveri*	35	15	9	7	11	43		**•**	**•**
15	*Saturnispora silvae*	31	9	6	16	13	47		**•**	
16	*Diutina catenulata*	25	16	4	7	5	35	**•**	**•**	**•**
17	*Candida baotianensis*^(***)^	24	12	9	11	7	43		**•**	
18	*Lachancea kluyveri*	24	9	6	9	8	33		**•**	
19	*Candida sake*^(***)^	23	12	6	3	5	27	**•**	**•**	
20	*Barnettozyma californica*	20	7	6	8	5	27		**•**	
21	*Meyerozyma guilliermondii*	20	9	3	8	3	23	**•**	**•**	**•**
22	*Saturnispora dispora*	17	11	4	9	4	33		**•**	
23	*Geotrichum sp.*	17	14	1	3		21			**•**
24	*Candida albicans*	17	7	6	5	2	20	**•**	**•**	**•**
25	*Candida solani*^(***)^	16	5	1	7	7	20		**•**	**•**
26	*Candida railensis*^(***)^	15	4	4	5	3	16		**•**	
27	*Yamadazyma scolyti*	14	5	3	7	2	18		**•**	
28	*Candida palmioleophila*	14	5	6	4	2	17	**•**	**•**	**•**
29	*Papiliotrema laurentii*^(***)^	14	1	1	10	3	17	**•**	**•**	**•**
30	*Kluyveromyces dobzhanskii*	13	7	5	4	3	23			**•**

*MSI-2*_*(i)*_ initial MSI-2 database, *MSI-2*_*(s)*_ supplemented MSI-2 database, *BRU* Bruker Library RUO

(^*^) *n*: number of isolated strains, using ^(1)^CH: CHROMagar Candida, ^(2)^DG: DG18, ^(3)^DR: DRBC, ^(4)^SY: SYMPHONY

(^**^) *n*_i_: number of isolates

(^***^) Species for which reference MALDI-TOF MS spectra have been added to the MSI-2 database

Ninety isolates (3.6%) remained unidentified at the end of the study. The lost strains (*n=65*) were mostly unidentifiable due to early overgrowth by filamentous fungi. The unknown strains (*n=25*), corresponding to strains that could not be identified using molecular barcoding, appeared to be evenly distributed among the four media (range from four to eight). These 90 isolates represented an undetermined number of species and were not considered in the evaluation of the performance of the media.

### Performance of the Media

**Fig. 1:**
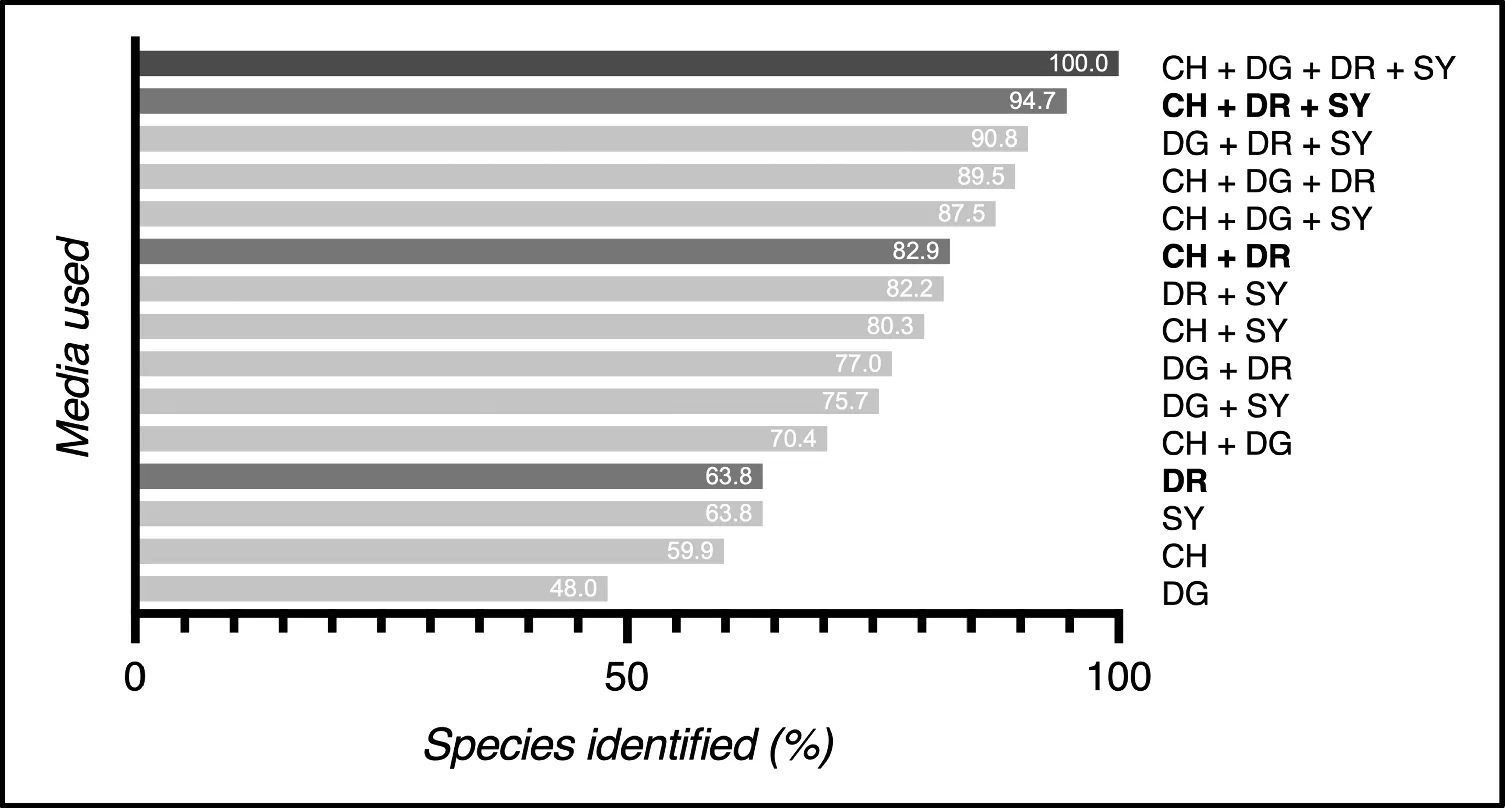
Percentage of species isolated on each medium or combination of media. (*CH*): CHROMagar Candida, (*DG*): DG18, (*SY*): SYMPHONY, (*DR*): DRBC

Evaluation of the media’s performance was based on the 152 species identified by the combination of the four media. An average of 90/152 species (58.9%) were found using only one culture medium for yeast identification. The DRBC and SYMPHONY media were the most effective, with 97 (63.8%) species potentially isolated, whereas the DG medium was the least effective, with 73 (48%) species isolated (Fig. 1). The average number of species using two media was 118/152 (78.1%), ranging from 107 to 126 (70.4–82.9%). Using a combination of three media was more effective, with an average of 138/152 (90.6%) species isolated, ranging from 133 to 144 (87.5–94.7%) (Table 2).

**Table 2:** Number of species and isolates identified for each medium and combination of media

Number of media	Media	Species		Isolates	
		*n*	(*%*)	*n*	(*%*)
1 medium	DG	73	(*48.0*)	556	(*22.4*)
	CH	91	(*59.9*)	659	(*26.6*)
	SY	97	(*63.8*)	565	(*22.8*)
	DR	97	(*63.8*)	699	(*28.2*)
*Mean*		*89.5*	(*58.9*)	*619.75*	*(25.0)*
2 media	CH + DG	107	(*70.4*)	1215	(*49.0*)
	DG + SY	115	(*75.7*)	1121	(*45.2*)
	DG + DR	117	(*77.0*)	1255	(*50.6*)
	CH + SY	122	(*80.3*)	1224	(*49.4*)
	DR + SY	125	(*82.2*)	1264	(*51.0*)
	CH + DR	126	(*82.9*)	1358	(*54.8*)
*Mean*		*118.7*	(*78.1*)	*1239.5*	(*50.0*)
3 media	CH + DG + SY	133	(*87.5*)	1780	(*71.8*)
	CH + DG + DR	136	(*89.5*)	1914	(*77.2*)
	DG + DR + SY	138	(*90.8*)	1820	(*73.4*)
	CH + DR + SY	144	(*94.7*)	1923	(*77.6*)
*Mean*		*137.8*	(*90.6*)	*1859.25*	(*75.0*)
4 media	CH + DG + DR + SY	152	(*100*)	2389	(*96.4*)
	Total	152	***100%***	2479	***100%***

*CH* CHROMagar Candida, *DG* DG18, *SY* SYMPHONY, *DR* DRBC

Using a four-media combination was significantly more efficient for species identification than using a single media (*p<0.0001*) or a combination of two media (*p=0.0009*). The four-media combination was more efficient than the three-media combinations, but the difference was not statistically significant (*p=0.1955*). The four-media combination also allowed a significantly higher number of isolates to be identified than either three-media (*p=0.0002*) or two-media (*p<0.0001*) combinations.

### Invasion of the Culture Media

**Fig. 2:**
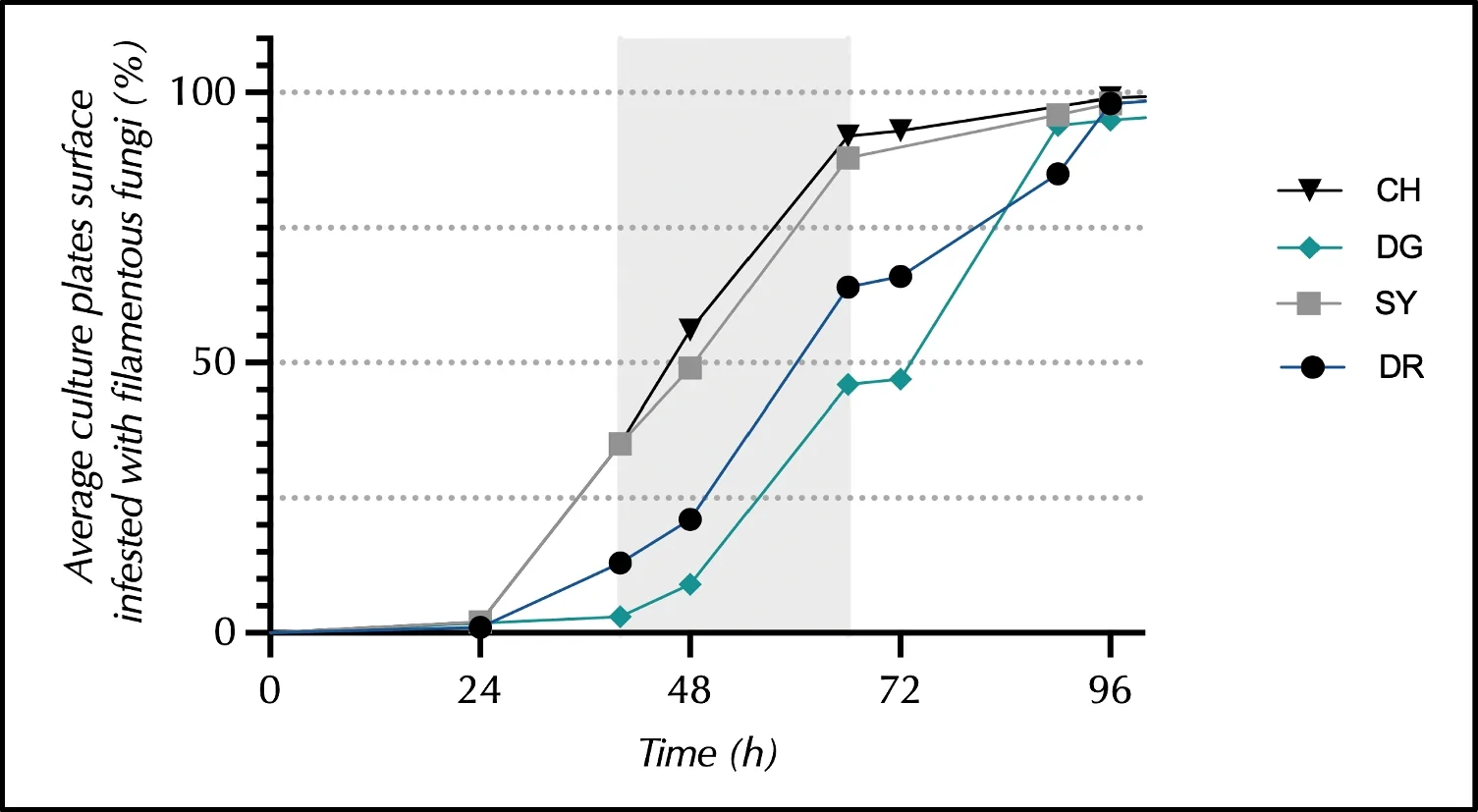
Percentages of the surface area of culture plates colonised by filamentous fungi over time. The grey area represents the time periods during which primary plates were subcultured. CH CHROMagar, DG DG18, SY SYMPHONY, DR DRBC

On average, 35% of the surface of the CHROMagar and SYMPHONY media was overgrown with filamentous fungi after 40 h of incubation versus 13% of the DRBC surface and only 3% of that of DG18. After 66 h of incubation, nearly 90% of the surface of the CHROMagar and SYMPHONY media was overgrown versus 65% of the DRBC surface and less than 50% of that of DG18. After 96 h of incubation, all media were considered to be 100% overgrown (Fig. 2).

### MALDI-TOF MS vs PCR

MALDI-TOF MS allowed the identification of 2119 isolates (88.7%), representing 113 of 152 species (74.3%). The rest of the isolates (*n*=270; 11.3%) were additionally identified by sequencing. They belonged to 88 different species, of which 39, representing 77 isolates, were identified only by sequencing analysis.

During the study, we added spectral data for 21 species to the MSI-2 database. Among them, 15 were new species that were missing from the original database. The other six were complementary spectra added to species already existing in the database. Six of these 21 species were among the 30 most common species that we identified (Table 1). At the end of our study, we compared the initial databases (June 2020) to those that had been supplemented (November 2021). In June 2020, 65 species of the 152 we identified during the study were identifiable with the initial MSI-2 database, corresponding to 62.7% of our isolates. After the addition of new species, the supplemented MSI-2 database (November 2021) could identify 95 species, corresponding to 88% of our isolates. The use of supplemented MSI-2 database combined with the Bruker library allowed the identification of 2283 isolates (92%) from 111 species (73%).

## Discussion

### Combining Culture Media Enhances the Biodiversity of Yeast Identified in Water

Culture-based methods are low-cost, practical in routine practise, and do not require a high level of expertise [5]. The use of multiple and diverse combinations of media has already been shown to increase the biodiversity of fungi found in water [10]. However, yeasts have rarely been studied specifically. This led us to investigate a strategy combining a medium specifically developed for yeasts (CHROMagar Candida), another nutrient-rich medium (Symphony), and two media containing dichloran, known to inhibit the growth of fast-growing moulds. We found the identification of yeast biodiversity in karst water to be significantly enhanced by using a combination of complementary media. While a single culture medium only detects 59% of biodiversity, combinations of media improve performance, detecting 78% and 91% of biodiversity, respectively with two or three media. The four-media combination was chosen as the standard with an arbitrary optimum performance of 100%. However, this does not reflect reality. While the four-environment strategy provides optimal biodiversity, we cannot be certain that all cultivable species have been isolated using this combination. The use of other additional media could have further increased biodiversity, reducing the relative performance of the four-media combination.

In this study, the combination of media enabled the coverage of a wide range of species due to their specific properties. Due to its chromogenic detection, CHROMagar Candida medium enhances the detection of yeast with specific enzymatic activity [18]. Hence, it facilitates the differentiation and isolation of yeast colonies on primary culture plates based on macromorphological criteria. CHROMagar medium is particularly efficient for the detection of *Candida* species, *Pichia* species, and *Rhodotorula* species [18, 19]. Indeed, we observed these species to be highly present in our karst water samples using this medium (Table 1). Thus, CHROMagar with DRBC was the best two-media combination for yeast recovery in our study. At least, using a chromogenic medium such as CHROMagar for the analysis of karst water avoids duplicate isolation of the same strains and is cost-effective while still allowing the detection of high yeast biodiversity.

The DG18 and DRBC media are selective for the identification of yeast by inhibiting filamentous fungi overgrowth due to the presence of dichloran. DRBC medium has been widely used for yeast identification in water samples [14, 20–23] since it was reported in the Swedish standard method for the analysis of micro-fungi in water [14]. According to the literature and our results, DRBC medium is the most efficient for the identification of high yeast biodiversity [21]. However, we found DG18 medium to be more efficient in inhibiting filamentous growth and its capacity to isolate xerophilic species and black yeast-like species of interest [24, 25]. The DG18 medium allowed us to isolate five of six strains of *Aureobasidium pullulans* group. Of note, MALDI-TOF MS database did not allow differential diagnosis between *A. pullulans* and its sister species *A. melanogenum*, previously described with high prevalence in Slovenian water [26]. Moreover, DG18 medium promoted growth of 32 out of 52 strains of the xerophilic species *Debaryomyces hansenii*. Although the DG18 medium showed the lowest identification rate in our study, it was still essential for the identification of certain species and enlarging the yeast biodiversity identified.

SYMPHONY medium has only been recently developed for food analysis. There are currently no studies in the literature on this medium. However, it appears to be promising for the analysis of yeast in water, as we found it to be as efficient as DRBC medium for yeast diversity identification (Fig. 1). It also allowed the identification of a strain belonging to the black-yeast *Aureobasidium pullulans* group. Further studies are needed to evaluate this medium in environmental studies.

### Supplementing and Combining the MALDI-TOF MS Databases Is Essential

MALDI-TOF MS has recently emerged as an alternative to conventional historical biochemical tests for the routine clinical identification of yeast. This transition has been attributed to its higher efficiency, rapid processing, and cost-effectiveness [27, 28]. Although MALDI-TOF MS is widely used in mycology laboratories for the identification of pathogenic species, its applicability to environmental species has been hampered by substantial gaps in existing databases. As a result, MALDI-TOF MS is largely underrepresented in studies of environmental yeast biodiversity. In the literature, the identification of environmental yeast species is mainly performed by molecular approaches based on PCR and sequencing targeting the ITS region [6, 29–32]. However, the use of MALDI-TOF MS allows significant savings in terms of both time and cost [33–35]. Consequently, adopting MALDI-TOF MS over PCR and sequencing methods offers the opportunity to analyse a substantially larger number of isolates.

Supplementing the databases with the spectra of missing species is essential to increasing the potential environmental applications of MALDI-TOF MS [36]. Our strategy consisted of supplementing the MSI-2 database because it has the advantage of being a collaborative and open access database. At the end of our study, the supplemented MSI-2 database was able to identify 88% of our isolates versus 63% at the beginning, and the number of identifiable species increased from 65 to 95 out of the 152 submitted. This is a significant improvement in the identification rate of environmental yeast biodiversity in water and demonstrates the importance of supplementing the databases. Furthermore, the addition of spectra from 21 species to the MSI-2 database allowed us to include 315 of our isolates, 123 belonging to the five species that were among the 30 most frequently observed. On the contrary, the updated Bruker library contained only three new species that we identified, representing 29 isolates. This demonstrates the effectiveness of supplementing a freely accessible database, such as MSI-2. However, the best way to improve the identification of environmental yeasts remains the combination of databases. Indeed, combining the MSI-2 database with the Bruker library allowed us to identify 111 species, representing 92% of our isolates. The databases are complementary: combining them improves the ability to identify more yeast species as well as the confidence level of identification. At last, adding new spectra from different strains of the same species improves the identification rate and provides better taxonomic identification scores [36].

A limitation of our study was that we compared the databases retrospectively. During the study, we were unable to supplement the MSI-2 database in real time. A few weeks elapsed between the molecular identification of a newly identified species and the acquisition of its spectra in the database. Therefore, we could only retrospectively estimate the performance of the database at the beginning of our study and compare it to the supplemented one at the end of our study.

### Importance of the Microbial Analysis of Drinking Water

Human-made environments, such as households and hospitals, are known to be major habitats for opportunistic fungi [37]. Groundwater from karst aquifers favours the transmission of fungal contaminants to drinking water, and numerous studies have identified pathogenic fungi in household water systems [5, 6, 31, 38, 39]. In our study, several opportunistic pathogenic yeast species were identified, such as *Candida parapsilosis*, *Rhodotorula mucilaginosa*, and *Exophiala dermatitidis* [40, 41]. These species have been reported to be emerging pathogens and have already been identified in groundwater and households, representing a source of human exposure. In addition, we found only several strains of the human pathogen *Candida albicans*, which may be an indicator of human contamination of drinking water sources. We also reported the high presence of *Nakaseomyces glabratus* (formerly *Candida glabrata*), the second most common human pathogen isolated from yeast fungemia [42]. Furthermore, we observed the high presence of *Pichia kudriavzevii* (formerly *Candida krusei*), which is intrinsically fluconazole resistant and also frequently associated with human fungemia. With an increasing immunosuppressed population, it is crucial to analyse yeast biodiversity in environmental sources of drinking water, such as karst waters. Our current knowledge of the biodiversity of environmental yeast species and their transmission by drinking water reveals the need to regulate their presence in drinking water. Thus, the development of a standard analytical protocol is crucial.

## Conclusion

In this study, our strategy was to improve the identification of yeast biodiversity in karst waters by developing an optimal culture-based method. The number of strains identified in our study was much higher than in any other study in the literature, which is an encouraging and promising result. Moreover, our work clearly demonstrates the importance of using complementary media to study the diversity of yeasts in water and justifies at least a combination of a nutrient-rich medium and a selective medium containing dichloran. The use of MALDI-TOF MS, combined with improved databases enriched with environmental strains, enables further larger-scale studies to be carried out at a lower cost. However, it only allows the species present in the base to be identified. The drawback of the MS databases justifies the complementary use of DNA barcoding to identify species that cannot be identified or distinguished using MALDI-TOF MS. As drinking water is an important source of human exposure to fungal contaminants, its analysis needs to continue. The analysis of yeast biodiversity in karst waters is a first step towards assessing the biosafety level of each fungal contaminant and establishing drinking water regulations. The next step will be to analyse many drinking water networks, identify their yeast biodiversity, and investigate the parameters that influence it.

## Supplementary Information

ESM 1 (PDF 557 KB)

## Data Availability

The datasets generated during and/or analysed during the current study are available from the corresponding author on reasonable request. Supplementary material associated with this article can be found in the online version.
