# Supplementary material for: Yeast Biodiversity of Karst Waters: Interest of Four Culture Media and an Improved MALDI-TOF MS Database
Source: Microb Ecol. 2024 Jan 4;87:26. doi: 10.1007/s00248-023-02336-1 (PMC10766713; doi:10.1007/s00248-023-02336-1)
Supplement: Supplementary file 1 [file 248_2023_2336_MOESM1_ESM.pdf]

IDENTIFICATION	n (strains)	n (isolates)	Added in MSI-2
Lost strains	-	65	
Unknown	-	25	
Pichia fermentans	103	246	
Metschnikowia pulcherrima	90	243	
Hanseniaspora uvarum	84	226	
Rhodotorula mucilaginosa	72	119	
Candida pseudolambica	57	87	X
Saccharomyces cerevisiae	52	93	
Pichia kudriavzevii	51	77	
Nakazawaea holstii	47	80	
Wickerhamomyces anomalus	42	66	
Nakaseomyces glabratus	42	65	
Debaryomyces hansenii	41	52	
Nakazawaea wyomingensis	36	52	
Clavispora lusitaniae	35	48	
Pichia kluyveri	35	43	
Saturnispora silvae	31	47	
Diutina catenulata	25	35	
Candida baotianensis	24	43	X
Lachancea kluyveri	24	33	
Candida sake	23	27	X
Barnettozyma californica	20	27	
Meyerozyma guilliermondii	20	23	
Saturnispora dispora	17	33	
Geotrichum sp	17	21	
Candida albicans	17	20	
Candida solani	16	20	X
Candida railensis	15	16	X
Yamadazyma scolyti	14	18	
Candida palmioleophila	14	17	
Papiliotrema laurentii	14	17	X
Kluyveromyces dobzhanskii	13	23	
Hanseniaspora valbyensis	13	16	
Candida norvegica	13	14	
Candida vartiovaarae	12	16	X
Nakazawaea ambrosiae	12	13	
Torulaspora delbrueckii	12	13	
Candida saitoana	11	15	X
Yamadazyma mexicana	11	15	X
Candida tetrigidarum	11	11	X
Meyerozyma caribbica	9	13	
Candida intermedia	9	10	
Kazachstania humilis	9	14	
Pichia occidentalis	8	10	
Cyberlindnera suaveolens	8	9	
Nakazawaea anatomiae	8	9	X
Scheffersomyces stipitis	8	8	

Pichia membranifaciens	*8*	*10*	
Candida parapsilosis	*7*	*13*	
Kuraishia molischiana	*7*	*10*	X
Candida oleophila	*7*	*9*	X
Candida boidinii	*7*	*8*	
Metschnikowia sp.	*7*	*12*	
Kazachstania exigua	*6*	*9*	
Debaryomyces nepalensis	*6*	*7*	X
Aureobasidium pullulans	*6*	*6*	
Candida tropicalis	*6*	*6*	
Kluyveromyces marxianus	*6*	*6*	
Candida santamariae	*5*	*6*	
Candida membranifaciens	*5*	*5*	
Candida zeylanoides	*5*	*5*	
Ogataea ramenticola	*5*	*5*	
Apiotrichum montevideense	*4*	*4*	
Wickerhamomyces canadensis	*4*	*4*	
Candida montana	*3*	*4*	
Cyberlindnera amylophila	*3*	*4*	
Hanseniaspora osmophila	*3*	*4*	
Kluyveromyces wickerhamii	*3*	*4*	
Wickerhamomyces bisporus	*3*	*4*	X
Candida sorboxilosa	*3*	*3*	X
Cutaneotrichosporon curvatum	*3*	*3*	
Hanseniaspora meyeri	*3*	*3*	
Hanseniaspora opuntiae	*3*	*3*	
Kurtzmaniella natalensis	*3*	*3*	
Metschnikowia sinensis	*3*	*3*	
Moesziomyces aphidis	*3*	*3*	
*Prototheca sp.**	*3*	*3*	
Cyberlindnera jadinii	*2*	*4*	
Hyphopichia paragotoi	*2*	*4*	
Candida trypodendri	*2*	*3*	
Coniochaeta mutabilis	*2*	*3*	
Cyberlindnera bimundalis	*2*	*3*	
Saccharomyces paradoxus	*2*	*3*	
Candida boleticola	*2*	*2*	
Candida cabralensis	*2*	*2*	
Candida melibiosica	*2*	*2*	
Candida sp.	*2*	*2*	
Cyberlindnera sp.	*2*	*2*	
Galactomyces geotrichum	*2*	*2*	
Komagataella pastoris	*2*	*2*	
Metschnikowia reukaufii	*2*	*2*	
Nakazawaea populi	*2*	*2*	X
Ogataea dorogensis	*2*	*2*	
Pichia manshurica	*2*	*2*	
Rhodosporidium sp	*2*	*2*	

Rhodotorula dairenensis	*2*	*2*	
Rhodotorula glutinis	*2*	*2*	
Rhodotorula sp.	*2*	*2*	
Wickerhamiella sorbophila	*2*	*2*	
Zygotorulaspora florentina	*2*	*2*	
Nakazawea sp.	*1*	*4*	
Trichosporon jirovecii	*1*	*3*	
Candida anglica	*1*	*2*	
Pichia cactophila	*1*	*2*	
Pichia exigua	*1*	*2*	
Trichosporon mucoides	*1*	*2*	
Apiotrichum laibachii	*1*	*1*	X
Apiotrichum mycotoxinivorans	*1*	*1*	
Barnettozyma populi	*1*	*1*	X
Barnettozyma vustinii	*1*	*1*	
Candida akabanensis	*1*	*1*	
Candida ethanolica	*1*	*1*	
Candida germanica	*1*	*1*	
Candida metapsilosis	*1*	*1*	
Candida orthopsilosis	*1*	*1*	
Candida schatavii	*1*	*1*	
Crinitomyces flavificans	*1*	*1*	
Cyberlindnera japonica	*1*	*1*	
Cyberlindnera saturnus	*1*	*1*	
Diutina rugosa	*1*	*1*	
Exophiala dermatitidis	*1*	*1*	
Filobasidium uniguttulatum	*1*	*1*	
Gibellulopsis sp.	*1*	*1*	
Hyphozyma variabilis	*1*	*1*	
Kazachstania gamospora	*1*	*1*	
Kazachstania pintolopesii	*1*	*1*	
Kazachstania servazzii	*1*	*1*	
Kazachstania wufongensis	*1*	*1*	
Kluyveromyces lactis	*1*	*1*	
Kuraishia capsulata	*1*	*1*	
Metschnikowia koreensis	*1*	*1*	
Millerozyma farinosa	*1*	*1*	
Naganishia albida	*1*	*1*	
Naganishia diffluens	*1*	*1*	
Naganishia liquefaciens	*1*	*1*	
Ogataea cecidiorum	*1*	*1*	
Pichia nakasei	*1*	*1*	
Scheffersomyces insectosa	*1*	*1*	
Schwanniomyces capriotti	*1*	*1*	X
Starmerella bacillaris	*1*	*1*	
Suhomyces ambrosiae	*1*	*1*	X
Suhomyces rilaensis	*1*	*1*	
Trichosporon asahii	*1*	*1*	

Trichosporon coremiiforme	*1*	*1*	
Trichosporon gracile	*1*	*1*	
Trichosporon japonicum	*1*	*1*	
Vanrija albida	*1*	*1*	
Wickerhamiella infanticola	*1*	*1*	
Wickerhamomyces onychis	*1*	*1*	
Wickerhamomyces silvicola	*1*	*1*	
Wickerhamomyces sydowiorum	*1*	*1*	
Yamadazyma sp.	*1*	*1*	
Yamadazyma terventina	*1*	*1*	
Yarrowia lipolytica	*1*	*1*	
**TOTAL**	**1485**	**2479**	

* Prototheca are algae that form yeast-like colonies.
